# Supplementary material for: An Integrated Mass Spectrometry-Based Glycomics-Driven Glycoproteomics Analytical Platform to Functionally Characterize Glycosylation Inhibitors
Source: Molecules. 2022 Jun 14;27(12):3834. doi: 10.3390/molecules27123834 (PMC9228227; doi:10.3390/molecules27123834)
Supplement: Supplementary file 1 [file molecules-27-03834-s001.zip › supplementary_molecules_04072022/molecules-1695691-supplementary.doc]

**An integrated mass spectrometry-based glycomics-driven glycoproteomics analytical platform to functionally characterize glycosylation inhibitors**

**Keywords:** glycomics/glycoproteomics/glycosylation/*in silico* docking/network pharmacology/non-small cell lung cancer/proteomics

Michael Russelle S. Alvarez^1,2^, Qingwen Zhou^2^, Sheryl Joyce B. Grijaldo^1^, Carlito B. Lebrilla^2^, Ruel C. Nacario^1^, Francisco M. Heralde III^3^, Jomar F. Rabajante^4^, Gladys C. Completo^1*^

^1^Institute of Chemistry, College of Arts and Sciences, University of the Philippines Los Baños, Laguna, Philippines

^2^Department of Chemistry, University of California Davis, Davis, California, U.S.A.

^3^Lung Center of the Philippines, Quezon City, Philippines

^4^Institute of Mathematical Sciences and Physics, College of Arts and Sciences, University of the Philippines Los Baños, Laguna, Philippines

Correspondence:

Gladys C. Completo, PhD

[gjcompleto@up.edu.ph](mailto:gjcompleto@up.edu.ph)

Institute of Chemistry, College of Arts and Sciences, University of the Philippines Los Baños, Laguna, Philippines

**Supplementary Files**

**Supplementary figures**

Supplementary figure S1. 24- and 48-hr dose-response toxicity of pictilisib.

Supplementary figure S2. Dose-response glycomics assay of pictilisib. [Add title]

Supplementary figure S3. Reproducibility of glycomics results between groups.

Supplementary figure S4. Reproducibility of proteomics results between groups.

Supplementary figure S5. –log10 FDR value results of underexpressed proteins by GSEA using Biological Process Gene Ontology set.

Supplementary figure S6. –log10 FDR value results of overexpressed proteins by GSEA using Biological Process Gene Ontology set.

Supplementary figure S7. Apoptosis assay results of pictilisib treatment.

Supplementary figure S8. Cell cycle assay results

Supplementary figure S9. Scratch assay results of pictilisib-treatment.

Supplementary figure S10. Transwell migration assay results of pictilisib-treatment.

Supplementary figure S11. Reproducibility of glycoproteomics results between groups.

Supplementary figure S12. Variation of protein glycosylation across all glycosites.

Supplementary figure S13. Pathways of proteins with reduced fucosylation, sialylation, or sialofucosylation.

Supplementary figure S14. Molecular function of proteins with reduced fucosylation, sialylation, or sialofucosylation.

**Supplementary tables**

Supplementary table S1. Mass spectrometry glycomics results showing relative abundances of each N-glycan.

Supplementary table S2. Mass spectrometry proteomicss results showing relative abundances of each protein.

Supplementary table S3. Mass spectrometry glycoproteomics results showing relative abundances of each glycopeptide.

Supplementary table S4. Mass spectrometry glycoproteomics results showing relative abundances of each protein glycosite.

Supplementary table S5. Mass spectrometry glycoproteomics results showing relative abundances of each integrin alpha-5 (ITGA5) and integrin beta-1(ITGB1) glycosylation.

Supplementary table S6. Molecular dynamics analysis of ITGA5-ITGB1 complex in vehicle control-treated cells.

Supplementary table S7. Molecular dynamics analysis of ITGA5-ITGB1 complex in pictilisib-treated cells.

Supplementary table S8. Predicted gene-drug interaction network of compound library against glycosylation genes.

Supplementary table S9. Predicted *in silico* binding energies of compound library against glycosylation enzymes.

**Supplementary videos**

Supplementary video S1. Molecular dynamics trajectories of ITGA5-ITGB1 complex in vehicle control-treated cells.

Supplementary video S2. Molecular dynamics trajectories of ITGA5-ITGB1 complex in pictilisib-treated cells.
